# Perinatal Risk Factors, Allergic Conditions and Psychosocial Problems in Children and Adolescents with Mental Health Problems

**DOI:** 10.1007/s10802-026-01465-8

**Published:** 2026-05-26

**Authors:** Sara Pieters, William J. Burk, Helen Klip, Carolina de Weerth, Malindi van der Mheen, Gabry W. Mies, Emilie M. A. van Tetering, Anne-Marie van den Hoek, Wendy J. M. A. van Summeren, Wouter Staal, Tinca J. C. Polderman

**Affiliations:** 1 Radboud University, Behavioural Science Institute, Thomas Van Aquinostraat 4, Nijmegen, 6525 GD the Netherlands; 2 Karakter, Child and Adolescent Psychiatry, Reinier Postlaan 12, Nijmegen, 6525 GC the Netherlands; 3 Radboud University Medical Center, Donders Institute for Brain, Cognition and Behaviour, Kapittelweg 29, Nijmegen, 6525 EN the Netherlands; 4 Department of Child and Adolescent Psychiatry, Amsterdam UMC, Meibergdreef 5, Amsterdam, 1105 AZ the Netherlands; 5 Department of Child and Adolescent Psychiatry & Psychosocial Care, Amsterdam UMC, Meibergdreef 5, 1105 AZ Amsterdam, the Netherlands; 6 Levvel, Academic Center for Child and Adolescent Psychiatry, Meibergdreef 5, Amsterdam, 1105 AZ the Netherlands; 7 Amsterdam Public Health Research Institute, Meibergdreef 9, Amsterdam, 1105 BK the Netherlands; 8 Kenniscentrum Kinder- en Jeugdpsychiatrie, Churchilllaan 11, Utrecht, 3527 GV the Netherlands; 9 Department of Psychiatry, Radboud University Medical Centre, Geert Grooteplein 18, Nijmegen, 6525 GA the Netherlands; 10 Leiden University, Wassenaarseweg 52, Leiden, 2333 AK the Netherlands; 11 Leiden University Medical Center Curium, Leiden, Postbus 15, 2300 AA the Netherlands; 12 Accare Child Study Center, Lübeckweg 2, Groningen, 9723 HE the Netherlands

**Keywords:** Allergic conditions, Perinatal risk factors, Youth, Mental health problems

## Abstract

Immune-related problems have been implicated in mental health disorders, yet their role in youth remains unclear. This study examined (a) the prevalence of perinatal risk factors and allergic conditions in youth receiving psychiatric care, (b) the association between these risks, and (c) their combined impact on psychosocial functioning. Participants included 2,193 children and adolescents (ages 6–17; *M* = 11.52, *SD* = 3.35). Parent-reported questionnaires assessed perinatal risks, allergic conditions, and psychosocial symptoms. Perinatal risks were reported in 31% and allergic conditions in 20% of the sample. No significant association emerged between the two risk domains. However, allergic conditions were linked to elevated internalizing symptoms. For externalizing symptoms, an effect of allergic conditions was only found in those with perinatal risks. Findings support the immuno-psychiatry framework, indicating that allergic conditions, particularly in combination with early-life risk, may contribute to psychosocial difficulties in clinically referred youth. Implications for early intervention are discussed.

**Supplementary Information:**

The online version contains supplementary material available at 10.1007/s10802-026-01465-8.

## Introduction

Immuno-psychiatry is an emerging field in psychiatry that examines the immune system as a mediator in the relationship between environmental stressors and the development of mental health conditions (Dantzer et al., 2008; Drexhage, 2023; Leboyer et al., 2016; Pariante, 2017). Specifically, it is proposed that environmental stressors (e.g., negative life events) induce detrimental biological effects that involve the immune system, which in turn increases vulnerability to psychiatric problems. Support for the immuno-psychiatry model comes from a myriad of studies, showing that early-childhood infections and auto-immune disorders are associated with an increased risk of psychiatric disorders, such as schizophrenia, autism spectrum disorder (ASD), and depression (Branchi et al., 2021; Dantzer et al., 2008; Hsiao, 2013; Jeppesen & Benros, 2019; Khandaker et al., 2015). Further, a familial history of auto-immune disorders has been shown to increase the risk of developing ASD, suggesting a shared genetic component (Chen et al., 2016). Likewise, a large genetic study on schizophrenia showed genetic overlap with immune-related diseases (Lam et al., 2019).

Several studies have indicated a higher prevalence of autoimmune diseases, such as type 1 diabetes, rheumatoid arthritis, psoriasis, and thyroid disorders, in adults with psychiatric disorders compared to healthy controls (Benros et al., 2011, 2014; Eaton et al., 2006). In children and adolescents, studies have also shown associations between immune-related processes and psychiatric disorders. Elevated levels of anti-neural antibodies are found in 30–70% of children with ASD (Ashwood & Van de Water, 2004; Van Gent et al., 1997), and a high prevalence of autoimmune diseases is documented in children with ASD (Chen et al., 2013), ADHD (Chen et al., 2017; Nielsen et al., 2017), and OCD (Arnold & Richter, 2001). Additionally, allergic conditions in early childhood have been linked to neurodevelopmental disorders (Chua et al., 2021). These findings emphasize the importance of a mind-body perspective in understanding the complex etiology of psychiatric disorders.

In line with the immuno-psychiatry hypothesis, adverse early-life events, including those during pregnancy and childbirth, may impact the developing immune system, potentially mediated by the gut microbiome (Mayer et al., 2015; Walker et al., 2017). This phenomenon, referred to as “early immune programming,” suggests that early immune dysregulation can have lasting developmental effects (Marques et al., 2013). Studies have shown that immune dysregulation in early development is linked to an increased risk of psychiatric disorders later in life (Brown & Meyer, 2018).

Prenatal factors, such as maternal immune activation (MIA) due to infections, have been associated with higher risks of neurodevelopmental disorders in offspring (Patel et al., 2021). Additionally, maternal prenatal stress has been linked to an increased risk of allergic conditions in children (Andersson et al., 2016; Suh et al., 2017). Furthermore, cesarean delivery, compared to vaginal delivery, has been associated with higher risks of allergies and autoimmune diseases later in life (Sevelsted et al., 2016). These findings suggest that immune dysregulation in response to early adversities may leave children vulnerable to negative spirals throughout development.

Although the relationship between immune-related conditions and mental health problems in children and adolescents has been studied before, the potential moderating role of perinatal risk factors on this relationship has not been studied extensively yet, to our knowledge. Furthermore, examining these relationships in a large heterogeneous (e.g., in terms of psychiatric problems or DSM-5 diagnoses) sample of youth registered for specialized mental health care provides a unique perspective on whether immune-related conditions might serve as a transdiagnostic factor in the complex etiology of psychiatric disorders.

The main aim of the current study was three-fold. First, we examined the prevalence of allergic conditions (asthma, food allergy, eczema, and hay fever) and perinatal risk factors (preterm birth, low birth weight, prenatal illness or infections, and Caesarian delivery) in a large sample of children and adolescents who are referred for specialized mental health care (aim 1). Second, we tested the potential relationship between perinatal risk factors and allergic conditions in these children and adolescents (aim 2). We hypothesized that youth with perinatal risk factors had a higher risk of experiencing allergic conditions than children and adolescents without perinatal risk factors. Third, we investigated the potential interaction between perinatal risk factors and allergic conditions on internalizing and externalizing problems (aim 3). We expected that the association between allergic conditions and internalizing and externalizing problems would be stronger in youth with perinatal risk factors compared to youth without perinatal risk factors.

## Method

### Participants

A total of 2,193 children and adolescents participated in this study, with a mean age of 11.52 years (*SD* = 3.35, range: 6–17 years; 51.2% boys). All children and adolescents were referred to a specialized center for inpatient and outpatient child and adolescent psychiatry in the Netherlands. In terms of DSM-5 diagnoses, the most prevalent diagnoses were ASD (46.6%), Attention-deficit hyperactivity disorder (41.1%), other neurodevelopmental disorders (15.7%), anxiety disorders (10.5%), trauma- or stressor-related disorders (8.4%), and mood disorders (7.4%). Diagnoses were made by licensed child and adolescents psychiatrists or clinical psychologists, in line with DSM-5, and were retrieved from electronic patient files for the current study. We included both primary and comorbid diagnoses in the frequency analysis.

### Procedure

Parents of youth registered for care in a specialized health care setting were invited to complete several questionnaires as part of routine care. For the current study, data were derived from questionnaires that were filled out by parents shortly before the intake appointment and from electronic patient files. Data collection took place between April 2021 and February 2023. Since parents completed these questionnaires prior to the intake appointment, no treatment at the specific center had been administered. Only the first record submitted by parents was used for data analysis (typically maternal reports). An opt-out procedure was used, whereby parents were informed digitally prior to intake and had the opportunity to refuse participation; data were only used for participants who did not opt out after completion of the questionnaires. This study was not subject to the Medical Research Involving Human Subjects Act. This was confirmed by the Medical Ethical Committee of Amsterdam University Medical Center (AMC; Van der Mheen et al., 2024). Furthermore, this study was approved by the Institutional Review Board of the specialized healthcare setting where the data were collected as part of routine practice.

### Materials

#### Intake Questionnaire

##### Allergic Conditions

Allergic conditions were measured using a single item from the intake questionnaire, phrased as “Does your child currently have any conditions or health problems?” Parents were asked to select relevant health conditions that pertain to their child from a list. For the current study, the response options “asthma”, “hay fever”, “eczema”, and “food allergy” were included. Other allergic or immune-related conditions were not assessed in this questionnaire. For the main analyses, these conditions were combined into a single binary variable indicating the presence of at least one condition. This grouping reflects a shared atopic/allergic background, consistent with common epidemiological practice (Arrieta et al. 2014a, b; Lowe et al. 2007; von Mutius, 2009), rather than clinical homogeneity. It should be noted that parent reports may reflect either a clinical diagnosis or parent-perceived symptoms.

##### Perinatal Risk Factors

Perinatal risk factors were assessed using four items from the intake questionnaire (see below for the description of the specific individual items). Perinatal risk factors consisted of preterm birth, low birth weight, maternal illness/infection during pregnancy (MIA), and caesarean section. These factors were combined into a single binary variable indicating the presence of at least one perinatal risk. This grouping reflects a set of biologically relevant exposures available in our dataset that may influence offspring immune and allergic development, consistent with prior epidemiological research (Arrieta et al. 2014a, b; Dominguez-Bello et al., 2010; Sandall et al., 2018; Stokholm et al., 2018). Absence of a reported risk does not imply absence of other prenatal risk factors, and the variable is not intended as a comprehensive measure of all perinatal adversity.

##### Pregnancy Duration

Pregnancy duration was assessed with the question: “How many weeks did the pregnancy last?”. Answer options were (1) “Less than 32 weeks (your child was born much too early, ‘extremely premature’)”, (2) “32–37 weeks (your child was born too early)”, (3) “37–42 weeks (your child was born after a normal-length pregnancy)” and (4) “42 weeks or more (your child was born after a pregnancy that was too long)”. An additional answer option (99) was added, reflecting the answer option “I don’t know”. Data from participants who selected this answer option were discarded from the main analyses. Answers were recoded such that a pregnancy duration shorter than 37 weeks was coded as 1 “preterm birth” and a duration of 37 weeks or longer was coded as 0 “normal pregnancy duration”.

##### Birth Weight

Gestational weight was assessed with the question: “How much did your child weigh at birth?”. Answer options were (1) “Less than 1,500 grams (extremely low birth weight)”, (2) “1,500-2,500 grams (low birth weight)”, (3) “2,500-4,000 grams (normal birth weight)”, and (4) “4,000 grams or more (high birth weight)”. Participants who selected the answer option (99) “I don’t know” were again discarded from the analyses. Responses were recoded such that a birth weight below 2500 g was coded as 1 “low birth weight” and a weight above 2500 g as 0 “normal birth weight”.

##### Prenatal Maternal Immune Activation

In response to the question: “Were there any particular problems during the pregnancy” one of the options was “Yes, illness or infections (e.g., measles, urinary tract infections, genital herpes, flu, jaundice)”. Participants who selected this answer option were considered to have experienced “prenatal maternal immune activation”, which was coded as 1. Participants who did not select this item were considered to have not experienced this type of (maternal) illness during pregnancy (coded as 0).

##### Delivery Mode

Delivery mode was assessed with the question: “Which of the following best describes your experience of childbirth?”. Options included (1) “Normal delivery”, (2) “Vacuum pump”, (3) “Forceps delivery” and (4) “Caesarian section”. Participants who selected the latter were coded as 1 “Caesarian section”, whereas all other answers were coded as 0 “no C-section”. Delivery mode was dichotomized as caesarean section versus vaginal delivery (including assisted vaginal delivery), based on literature linking caesarean birth to altered early microbial colonization and immune development (Arrieta et al. 2014a, b; Dominguez-Bello et al. 2010).

#### Child Behavior Checklist

##### Psychosocial Problems

Psychosocial problems were measured using the Dutch translation of the Child Behavior Checklist (CBCL)/6–18. The CBCL/6–18 is part of the Achenbach System of Empirically Based Assessment (ASEBA; Achenbach & Rescorla, 2014; Achenbach & Verhulst, 2010), which consists of several questionnaires aimed at measuring skills and problem behavior in children and adolescents aged 6–18 years. The CBCL/6–18 contains 120 items and is completed by parents and includes several subscales examining internalizing and externalizing problems as well as other problem behaviors displayed by the child or adolescent in the past six months. Internalizing problems are defined as emotional or other internal problems, including anxiety, depression and somatic complaints without a clear physical cause. Externalizing problems are defined as behavioral problems, including having conflicts with others, being aggressive and antisocial behavior. All items are answered on a three-point scale with answer options (0) “Not at all”, (1) “A bit or sometimes” and (2) “Clearly or Often”. The internalizing problems composite score is made up of the subscales “Withdrawn-Depressed” (13 items), “Anxious-Depressed” (8 items) and “Somatic Complaints” (11 items). The externalizing problems composite score is made up of the “Rule-breaking Behavior” (17 items) and the “Aggressive Behavior” (18 items) subscales. Internalizing and externalizing problems were the outcome measures that were used in the current study. Higher scores reflect more internalizing or externalizing problems. The CBCL/6–18 demonstrated excellent internal consistency in the current sample (α = .94 for the total scale), consistent with previous Flemish research (Braet et al., 2011). Evidence for construct validity is supported by studies showing replication of the CBCL syndrome structure in Dutch samples (De Groot et al., 1994). In addition, Braet et al. (2011) showed substantial correlations with the SDQ (approximately *r* = .60–.80 depending on scale), indicating good concurrent validity in Flemish populations.

### Data analysis

First, descriptive statistics were calculated to estimate the prevalence of allergic conditions and perinatal risk factors among children and adolescents with psychiatric problems, addressing the first aim of the study (aim 1). To test the study’s second hypothesis, concerning the potential relationship between perinatal risk factors (binary) and allergic conditions (binary), we conducted a logistic regression analysis controlling for age and sex (aim 2). Finally, two Analyses of Variance (ANOVAs) were performed to examine the effects of sex, allergic conditions, perinatal risk factors, and their interactions, on internalizing and externalizing problems, with age included as a covariate (aim 3). Two outliers (Z > 3) were detected on the internalizing problems outcome and 10 outliers (Z > 3) were detected on the externalizing problems outcome. Outliers were truncated to the highest score that was not defined as an outlier, before running the analyses. No outliers were found on the other variables. Effect sizes were calculated where appropriate and interpreted according to conventional benchmarks (Cohen, 2013).

To further examine the robustness of our findings and to explore both cumulative and individual contributions of risk factors, sensitivity analyses were conducted using cumulative indices of allergic conditions (range 0–4) and perinatal risk factors (range 0–4), as well as individual risk factors, for both internalizing and externalizing problems. In addition, quadratic terms and interactions between cumulative allergic burden and cumulative perinatal risk factors were included in the sensitivity analyses to examine potential non-linear and combined effects on internalizing and externalizing outcomes.

## Results

### Descriptive statistics

#### Allergic conditions (aim 1)

In Table 1, prevalence estimates for allergic conditions are presented separately for boys and girls. Overall, one in five children and adolescents with psychiatric problems experienced at least one allergic condition. Hay fever was the most prevalent condition, followed by eczema, asthma, and food allergies. No sex differences were observed in the prevalence of any allergic conditions. Additionally, no age differences were found between children and adolescents with and without allergic conditions (*t*(2155) = -1.14, *p* = .255, *d* = − 0.06).

**Table 1:** Prevalence of allergic conditions and perinatal risk factors in youth with psychiatric problems

	Boys(*n*= 1109)	Girls(*n* 1048)	Total(*N*= 2157)	Tests of sex differences	
*Allergic conditions*	*n (%)*	*n (%)*	*n (%)*	*χ*^2^(1) =	*p =*
Asthma	55 (5.0)	39 (3.7)	94 (4.4)	1.98	.159
Hay-fever	126 (11.4)	112 (10.7)	238 (11.0)	.25	.617
Eczema	84 (7.6)	71 (6.8)	155 (7.2)	.52	.472
Food allergy	47 (4.2)	46 (4.4)	93 (4.3)	.03	.863
Any of the specific allergic conditions	238 (21.5)	198 (18.9)	436 (20.2)	2.20	.138
	Boys(*n*= 1036)	Girls(*n*= 992)	Total(*N*= 2028)	Tests of sex differences	
*Perinatal risk factors*	*n (%)*	*n (%)*	*n (%)*	*χ*^2^(1) =	*p =*
Prenatal illness/infections	28 (2.7)	33 (3.3)	61 (3.0)	.68	.411
C-section	181 (17.5)	161 (16.2)	342 (16.9)	.54	.462
Preterm birth	154 (15.0)	122 (12.4)	276 (13.7)	2.83	.092
Low birth weight	128 (12.7)	123 (12.6)	251 (12.6)	.00	.976
Any of the specific perinatal risk factors	326 (32.3)	288 (29.8)	614 (31.1)	1.44	.231
	Boys(*n*= 989)	Girls(*n*= 929)	Total(*N*= 1918)	Tests of sex differences	
*Psychosocial problems*	*M (SD)*	*M (SD)*	*M (SD)*	*t(1916) =*	*p =*
Internalizing problems	16.49 (9.87)	23.58 (11.46)	19.93 (11.24)	-14.54	< .001
Externalizing problems	16.57 (10.78)	13.43 (9.92)	15.05 (10.49)	6.64	< .001

#### Perinatal Risk Factors (aim 1)

Prevalence estimates for perinatal risk factors are also shown in Table 1. Almost one-third of (the mothers of) children and adolescents reported experiencing at least one of these risk factors. The most commonly reported was C-section, followed by preterm birth and prenatal illness or infections. No sex differences were observed. The children of parents who reported at least one of the perinatal risk factors (*M* = 10.99, *SD* = 3.34) were on average younger compared to those who did not experience these perinatal risk factors (*M* = 11.72, *SD* = 3.31; *t*(1975) = 4.51, *p* < .001, *d* = 0.22), indicating a small effect.

#### Psychosocial Problems

Descriptive statistics for psychosocial problems are shown in Table 1. Mean scores showed that parents of girls reported more internalizing problems in their offspring and fewer externalizing problems, compared to parents of boys. The effect of sex was medium for internalizing problems (*d* = − 0.66) and small for externalizing problems (*d* = 0.30). Age was positively associated with internalizing problems (*r* = .363, *p* < .001), meaning that parents of older participants reported more internalizing problems than parents of younger participants. In contrast, age was negatively related to externalizing problems (*r* = − .131, *p* < .001), such that parents of younger participants reported more externalizing problems than parents of older participants.

### The Association between Perinatal Risk Factors and Allergic Conditions (aim 2)

To test the association between perinatal risk factors (binary) and allergic conditions (binary), a logistic regression analysis was conducted, with sex, age, and perinatal risk factors (and all two-way interactions) as independent variables and allergic conditions as dependent variable. Results showed that perinatal risk factors were not associated with allergic conditions (*Exp(B)* = 1.30, *95%CI Exp(B)* = [0.81-2.07], *p* = .272). All other main and interaction effects were also not statistically significant.

### The Interaction between Perinatal Risk Factors and Allergic Conditions on Internalizing and Externalizing Problems (aim 3)

Two Analyses of Variance (ANOVAs) were conducted with sex, allergic conditions and perinatal risk factors, and their interactions, as independent variables. Age was included as a covariate.

**Fig. 1:**
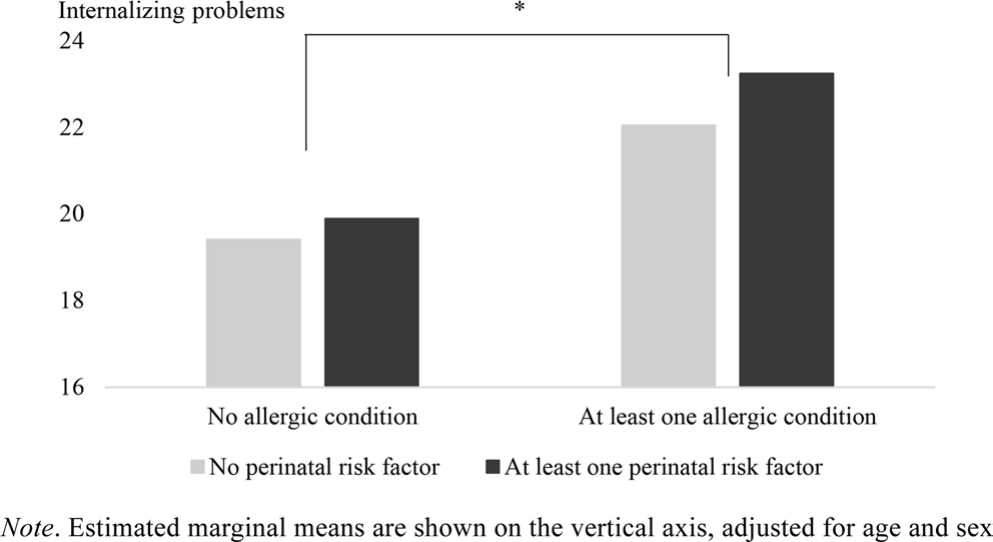
The association between perinatal risk factors, allergic conditions and internalizing problems: a main effect of allergic conditions

Results indicated a significant effect of allergic conditions on internalizing problems (*F*(1, 1768) = 20.31, *p* < .001, *η*^*2*^ = 0.01), indicating a small effect size. Parents who reported that their child had an allergic condition reported that their child exhibited more internalizing problems compared to parents not reporting that their child had an allergic condition (see Fig. 1).

There were no mean-level differences in parents’ reports of internalizing problems for those reporting at least one perinatal risk factor compared to those not reporting any perinatal risk factors (*F*(1, 1768) = 1.59, *p* = .207, *η*^*2*^ = 0.00). The interaction between perinatal risk factors and allergic conditions was not statistically significant (*F*(1, 1768) = 0.29, *p* = .590, *η*^*2*^ = 0.00). Main effects of sex (*F*(1, 1768) = 58.03, *p* < .001, *η*^*2*^ = 0.03) and age (*F*(1, 1768) = 162.11, *p* < .001, *η*^*2*^ = 0.08) were significant, indicating girls (*M* = 23.67, *SE* = 0.49) experienced more internalizing problems than boys (*M* = 18.62, *SE* = 0.45; small effect size), and that an older age was associated with more internalizing problems (*b* = 0.98; medium effect size). Interactions with sex were not significant.

The model with externalizing problems as an outcome did not reveal significant main effects of allergic conditions (*F*(1, 1768) = 1.79, *p* = .181, *η*^*2*^ = 0.00) or perinatal risk factors (*F*(1, 1768) = 2.41, *p* = .121, *η*^*2*^ = 0.00), but did show a significant interaction between perinatal risk factors and allergic conditions (*F*(1, 1768) = 4.03, *p* = .045, *η*^*2*^ = 0.00; small effect size). Main effects of sex (*F*(1, 1768) = 7.16, *p* = .008, *η*^*2*^ = 0.00) and age (*F*(1, 1768) = 14.10, *p* = < 0.001, *η*^*2*^ = 0.01) were significant, meaning that boys (*M* = 16.07, *SE* = 0.45) displayed more externalizing problems than girls (*M* = 14.30, *SE* = 0.49), and an older age was associated with fewer externalizing problems (*b* = − 0.29). Interactions with sex were not significant.

**Fig. 2:**
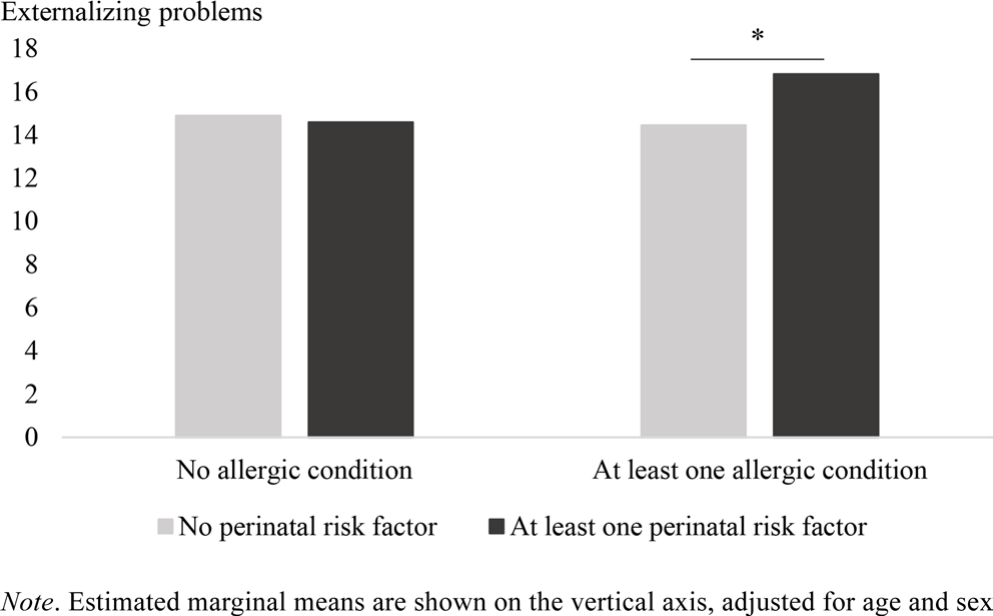
The association between perinatal risk factors, allergic conditions and externalizing problems: interaction effect between perinatal risk factors and allergic conditions

Tests of simple effects showed that a significant difference in externalizing problems between children and adolescents with and without allergic conditions was only detectable in those with at least one perinatal risk factor (*F*(1, 544) = 0.15, *p* = .042, *η*^*2*^ = 0.01; small effect size) and not in those without perinatal risk factors (*F*(1, 1222) = 0.33, *p* = .568, *η*^*2*^ = 0.00). For children whose parents reported perinatal risk factors, more externalizing problems were reported for those who also had an allergic condition compared to those who had not. Estimated marginal means for the model with externalizing problems as dependent variable are shown in Fig. 2.

### Sensitivity Analyses (Cumulative and Individual Risk Factors)

To assess the robustness of our primary findings, we conducted sensitivity analyses using cumulative indices of allergic conditions (range 0–4) and perinatal risk factors (range 0–4), as well as individual risk factors, for both internalizing and externalizing problems.

#### **Internalizing problems**

Higher cumulative allergic burden was significantly associated with higher internalizing scores, whereas cumulative perinatal risk was not significant (*F*(7, 1768) = 59.51, *p* < .001, Adjusted *R*^*2*^ = 0.19; see Supplementary Table 1). Quadratic terms and the interaction between cumulative allergic conditions and perinatal scores were non-significant, indicating a linear dose–response effect.

Examining individual risk factors revealed that eczema and food allergy were significantly associated with higher internalizing scores (*F*(10, 1765) = 42.71, *p* < .001, Adjusted *R*^*2*^ = 0.19; see Supplementary Table 2). Among individual perinatal risk factors, only maternal prenatal illness or infections reached significance, whereas all other individual predictors were non-significant.

#### **Externalizing Problems**

In contrast, cumulative indices did not show significant linear main or interaction effects for externalizing scores, and quadratic terms were non-significant, suggesting that a dose–response pattern is absent (*F*(7, 1768) = 8.20, *p* < .001, *R²* = 0.03; see Supplementary Table 3). However, it must be noted that the distribution of cumulative allergic conditions was highly skewed, with the majority of participants reporting no allergic conditions (79.8%), followed by one condition (14.8%), two conditions (4.2%), three conditions (1.2%), and four conditions (< 0.1%). A similarly skewed distribution was observed for cumulative perinatal risk factors, with most participants reporting no perinatal risk factors (68.9%), followed by one risk factor (19.5%), two risk factors (7.7%), three risk factors (3.6%), and four risk factors (0.2%). Therefore, the results of the cumulative analyses should be taken with caution.

Furthermore, analyses showed that none of the individual allergic conditions were significantly associated with externalizing problems, and that among the individual perinatal factors, only maternal prenatal illness or infections was significant (*F*(10, 1765) = 6.47, *p* < .001, *R*^*2*^ = 0.03; see Supplementary Table 4). This confirms that the threshold-like interaction observed in the primary binary analysis is not attributable to any single risk factor, but rather reflects the combination of allergic conditions and perinatal risk factors.

Overall, these sensitivity analyses demonstrate that the main findings are robust: internalizing problems show a linear dose–response with cumulative allergic burden, whereas externalizing problems are related to the presence of combined risk factors rather than cumulative linear effects.

## Discussion

The aims of the current study were threefold. The first aim was to establish in a large group of children and adolescents with psychiatric problems, prevalence estimates of parent-reported allergic conditions and perinatal risk factors. The second aim was to examine the relationship between allergic conditions and perinatal risk factors. Third, we aimed to explore the interaction between allergic conditions and perinatal risk factors on internalizing and externalizing problem behaviors in this population.

We found that approximately one in five children and adolescents with psychiatric problems experienced at least one allergic condition. Hay fever was found to be the most common allergic condition, followed by eczema, asthma, and food allergies. These prevalence estimates align with results of the National Health Interview Survey (NHIS) study, which examined diagnosed allergic conditions in a large sample of non-institutionalized American children and adolescents (Zablotsky et al., 2023). Findings are also consistent with other studies reporting prevalence estimates of hay fever, eczema, asthma and food allergies in children and adolescents from the general population (García-Marcos et al., 2022; Thamm et al., 2018). Taken together, these findings suggest that allergies are equally prevalent in children and adolescents from the general population compared to those with psychiatric problems.

We further found that approximately one-third of children and adolescents with psychiatric problems experienced at least one perinatal risk factor, such as prenatal maternal infections or illness (3.0%), Caesarean Sect.  (16.9%), preterm birth (13.7%), or low birth weight (12.6%). These findings appear to be partly in line with figures from the general population. A large Dutch study found preterm birth rates ranging from 5.2% for singletons to 54.0% for multiples (Klumper et al., 2024), while an international study reported around 10% of births as preterm (Ohuma et al., 2023). UNICEF-WHO estimates indicate that low birth weight occurs in roughly one in seven births (WHO, 2021). Prevalence rates for Caesarean sections are similar to or slightly lower than those reported in the general population, although there are large global differences (Betran et al., 2021; WHO, 2021). We note that assisted vaginal delivery may be associated with other adverse outcomes; however, our operationalization was theory-driven and focused specifically on hypothesized microbiota-related mechanisms relevant to allergic conditions.

Age-related effects were observed. These effects may reflect either a cohort effect or potential memory bias. Parents might forget certain events, particularly as their child reaches late adolescence. This issue is especially relevant for low-prevalence exposures, such as prenatal maternal illness or infections. Parents may not recall these events if they perceived the illness as trivial (e.g., a common flu) or if the mother experienced multiple pregnancies. In summary, prevalence estimates for perinatal risk factors generally align with those in the general population, although preterm birth rates may be higher than typical population estimates.

We found no evidence for a direct relationship between perinatal risk factors and allergic conditions. Consistent with the immuno-psychiatry hypothesis, we had hypothesized that early-life stressors, operationalized as perinatal risk factors, would be related to subsequent immune-related outcomes, including allergic conditions. The absence of a significant bivariate association in our data may reflect several methodological and conceptual considerations. First, some perinatal exposures were of low prevalence (e.g., maternal illness/infection), which may have limited statistical power to detect associations (Stokholm et al., 2018). Second, postnatal environmental factors, such as microbiota composition, diet, and early-life infections, as well as genetic susceptibility, may moderate or obscure the relationship between perinatal risk and allergic outcomes (Arrieta et al. 2014a, b; Dominguez-Bello et al. 2010). Third, retrospective parent-reported perinatal risk factors may be subject to recall bias, particularly for mild or subclinical exposures (Knopik, 2009). Finally, the cross-sectional and observational design of this study precludes causal inference. Longitudinal research is warranted to elucidate dynamic relationships between perinatal exposures and immune/allergic outcomes across development. Instead, we found a trend for an interaction effect between these two variables with respect to externalizing problems, and a main effect of allergic conditions on internalizing problems. Sensitivity analyses using cumulative indices and individual risk factors supported these patterns: internalizing problems showed a linear association with cumulative allergic burden, whereas externalizing problems were affected primarily by the combined presence of allergic conditions and perinatal risk factors rather than by cumulative linear effects. These findings align with previous research in children and adolescents from the general population or with specific immune-related conditions, which have shown a link between allergic conditions and emotional and behavioral problems (Chang et al., 2013; Hu et al., 2020); Nanda et al., 2016; Verkleij, Van de Griendt, Kaptein, Van Essen-Zandvliet, Duiverman, & Geenen, 2011). Furthermore, our results are consistent with previous studies in children and adolescents with psychiatric problems. Some of these studies explicitly focused on the relationship between immune-related problems and internalizing problems (Infante et al., 2007). The association with externalizing problems was examined to a lesser extent. In addition, no studies to date have examined the interaction with early risk factors in the pre- or perinatal period. Taken together, these findings may suggest that early risk factors related to the development of the immune system might interact with, or contribute to sensitivity for, the development of psychiatric problems. This interpretation is consistent with the immuno-psychiatry hypothesis.

Although this study has several strengths, including the large sample of youth with mental health problems, it also has several limitations. First, cross-sectional data were used, so the causal order of events cannot be established. Although we expected pre- and perinatal risk factors to predict subsequent internalizing and externalizing problems, specific statements on causality cannot be made. This also applies to the association between allergic conditions and psychosocial outcomes. While we hypothesized the effect of allergic conditions on psychosocial problems, it is also possible that psychosocial problems influence allergic conditions. Furthermore, another developmental vulnerability may underlie the observed associations, creating a negative spiral in development and a complex interplay between pre- and perinatal risk factors, allergic conditions, and psychosocial problems.

A second limitation concerns the measurement of study variables. All constructs were assessed via parent-report questionnaires. Prevalence estimates of allergic conditions may be biased, as it is unclear whether these reflect parental interpretation of symptoms or professional diagnoses. Diagnosing allergic conditions in children can be challenging, even for professionals; for instance, asthma may be difficult to distinguish from other respiratory disorders (Miravitlles et al., 2012). Therefore, prevalence estimates should be interpreted with caution. The same applies to pre- and perinatal risk factors, which may be subject to memory bias. Parents might forget certain events, or early-life events might be perceived differently due to current family challenges. In addition, it would have been relevant to consider whether parents themselves had allergies, given the high heritability of these conditions (Willemsen et al., 2008). Finally, the perinatal risk factors and allergic conditions included in this study represent a limited subset of potential exposures. Other relevant prenatal or perinatal influences, such as maternal autoimmune conditions, psychosocial stress, or substance use, were not assessed, which limits the generalizability of our findings. Future studies might benefit from more sophisticated research designs, such as longitudinal cohort studies, that allow better insight into potential negative spirals of immune-behavior crosstalk throughout development. Furthermore, other measurements should be included as well, such as those tapping physiological processes related to the immune system, to gain a better understanding of the underlying mechanisms.

In addition, it would be interesting to examine whether modifiable factors, such as lifestyle factors, moderate the relationship between immune-related problems and psychosocial problems, thereby informing potential avenues for intervention in this population. An interesting factor in this regard is sleep, due to its intricate relationships with both the immune system (Besedovsky et al., 2012; Majde & Krueger, 2005) and psychosocial problems (Pieters et al., 2015; Pieters, Van der Vorst, Burk, Wiers, & Engels, 2010; Van Tetering et al., 2024). In addition, sleep has been proposed as a crucial factor in neurodevelopment (Kurth et al., 2015). Despite the above-mentioned limitations, this is one of the first studies examining the interaction between perinatal risk factors and allergic conditions on both internalizing and externalizing problems in a large clinically referred sample of children and adolescents. As such, the findings may not be generalizable to population-based cohorts. The results of the current study add to the existing knowledge on the immune-psychiatry hypothesis, suggesting that a mind-body perspective in the assessment and treatment of psychiatric disorders is of great importance.

## Electronic Supplementary Material

Below is the link to the electronic supplementary material.

Supplementary Material 1

## Data Availability

The data for this study are not publicly available due to legal and ethical restrictions. The dataset contains pseudo-anonymized information from children and adolescents with complex psychiatric conditions receiving clinical care. Because of the sensitive nature of the data and the potential for indirect identification, even when anonymized, public access is not possible. In addition, an opt-out consent procedure was employed (see Methods), further limiting data sharing. These restrictions comply with institutional policies. Researchers with well-justified scientific purposes may request access by contacting the corresponding author.

## Declarations

### Preregistration statement

This research was not preregistered.

## References

[CR1] Achenbach, T. M., & Rescorla, L. A. (2014). The Achenbach system of empirically based assessment (ASEBA) for ages 1.5 to 18 years. *The use of psychological testing for treatment planning and outcomes assessment* (pp. 179–213). Routledge.

[CR2] Achenbach, T. M., & Verhulst, F. (2010). Achenbach system of empirically based assessment (ASEBA).

[CR3] Andersson, N. W., Hansen, M. V., Larsen, A. D., Hougaard, K. S., Kolstad, H. A., & Schlünssen, V. (2016). Prenatal maternal stress and atopic diseases in the child: A systematic review of observational human studies. *Allergy*, *71*(1), 15–26. 10.1111/all.12762 26395995 10.1111/all.12762 PMC5054838

[CR6] Arnold, P. D., & Richter, M. A. (2001). Is obsessive–compulsive disorder an autoimmune disease? *Canadian Medical Association Journal*, *165*(10), 1353–1358. 11760984 PMC81631

[CR4] Arrieta, M. C., Stiemsma, L. T., Amenyogbe, N., Brown, E. M., & Finlay, B. (2014a). The intestinal microbiome in early life: Health and disease. *Frontiers in Immunology*, *5*, 427. 10.3389/fimmu.2014.00427 25250028 10.3389/fimmu.2014.00427 PMC4155789

[CR5] Arrieta, M. C., Stiemsma, L. T., Dimitriu, P. A., Thorson, L., Russell, S., Yurist-Doutsch, S., & Finlay, B. (2014b). Early infancy microbial and metabolic alterations affect risk of childhood asthma. *Science Translational Medicine*, *6*(257), 257ra142. 10.1126/scitranslmed.3008892 10.1126/scitranslmed.aab2271 26424567

[CR7] Ashwood, P., & Van de Water, J. (2004). Is autism an autoimmune disease? *Autoimmunity Reviews*, *3*(7–8), 557–562. 10.1016/j.autrev.2004.07.036 15546805 10.1016/j.autrev.2004.07.036

[CR8] Benros, M. E., Nielsen, P. R., Nordentoft, M., Eaton, W. W., Dalton, S. O., & Mortensen, P. B. (2011). Autoimmune diseases and severe infections as risk factors for schizophrenia: a 30-year population-based register study. *American Journal of Psychiatry*, *168*(12), 1303–1310. 10.1176/appi.ajp.2011.11030516 22193673 10.1176/appi.ajp.2011.11030516

[CR9] Benros, M. E., Pedersen, M. G., Rasmussen, H., Eaton, W. W., Nordentoft, M., & Mortensen, P. B. (2014). A nationwide study on the risk of autoimmune diseases in individuals with a personal or a family history of schizophrenia and related psychosis. *American Journal of Psychiatry*, *171*(2), 218–226. 10.1176/appi.ajp.2013.13010086 24129899 10.1176/appi.ajp.2013.13010086

[CR10] Besedovsky, L., Lange, T., & Born, J. (2012). Sleep and immune function. *Pflügers Archives*, *463*(1), 121–137. 10.1007/s00424-011-1044-0 22071480 10.1007/s00424-011-1044-0 PMC3256323

[CR11] Betran, A. P., Ye, J., Moller, A. B., Souza, J. P., & Zhang, J. (2021). Trends and projections of caesarean section rates: global and regional estimates. *BMJ Global Health*, *6*(6), e005671. 10.1136/bmjgh-2021-005671 34130991 10.1136/bmjgh-2021-005671 PMC8208001

[CR12] Braet, C., Callens, J., Schittekatte, M., Soyez, V., Druart, C., & Roeyers, H. (2011). Assessing emotional and behavioural problems with the Child Behaviour Checklist: Exploring the relevance of adjusting the norms for the Flemish community. *Psychologica Belgica*, *51*(3–4), 185–202. 10.5334/pb-51-3-4-185

[CR13] Branchi, I., Poggini, S., Capuron, L., Benedetti, F., Poletti, S., Tamouza, R., & Pariante, C. M. (2021). Brain-immune crosstalk in the treatment of major depressive disorder. *European Neuropsychopharmacology*, *45*, 89–107. 10.1016/j.euroneuro.2020.11.016 33386229 10.1016/j.euroneuro.2020.11.016

[CR14] Brown, A. S., & Meyer, U. (2018). Maternal immune activation and neuropsychiatric illness: a translational research perspective. *American Journal of Psychiatry*, *175*(11), 1073–1083. 10.1176/appi.ajp.2018.17121393 30220221 10.1176/appi.ajp.2018.17121311 PMC6408273

[CR15] Chang, H. Y., Seo, J. H., Kim, H. Y., Kwon, J. W., Kim, B. J., Kim, H. B., & Hong, S. J. (2013). Allergic diseases in preschoolers are associated with psychological and behavioural problems. *Allergy Asthma Immunology Research*, *5*(5), 315–321. 10.4168/aair.2013.5.5.315 24003389 10.4168/aair.2013.5.5.315 PMC3756179

[CR16] Chen, M. H., Su, T. P., Chen, Y. S., Hsu, J. W., Huang, K. L., Chang, W. H., & Bai, Y. M. (2013). Comorbidity of allergic and autoimmune diseases in patients with autism spectrum disorder: A nationwide population-based study. *Research in Autism Spectrum Disorders*, *7*(2), 205–212. 10.1016/j.rasd.2012.08.0082015.08.035

[CR18] Chen, S. W., Zhong, X. S., Jiang, L. N., Zheng, X. Y., Xiong, Y. Q., Ma, S. J., Qiu, M., Huo, S. T., Ge, J., & Chen, Q. (2016). Maternal autoimmune diseases and the risk of autism spectrum disorders in offspring: a systematic review and meta-analysis. *Behavioural Brain Research*, *296*, 61–69. 10.1016/j.bbr.2015.08.035 26327239 10.1016/j.bbr.2015.08.035

[CR17] Chen, M. H., Su, T. P., Chen, Y. S., Hsu, J. W., Huang, K. L., Chang, W. H., & Bai, Y. M. (2017). Comorbidity of allergic and autoimmune diseases among patients with ADHD: a nationwide population-based study. *Journal of Attention Disorders*, *21*(3), 219–227. 10.1177/1087054712474686 23400216 10.1177/1087054712474686

[CR19] Chua, R. X. Y., Tay, M. J. Y., Ooi, D. S. Q., Siah, K. T. H., Tham, E. H., Shek, L. P. C., & Loo, E. X. L. (2021). Understanding the link between allergy and neurodevelopmental disorders: a current review of factors and mechanisms. *Frontiers in Neurology*, *11*, 603571. 10.3389/fneur.2020.603571 33658968 10.3389/fneur.2020.603571 PMC7917177

[CR61] Cohen, J. (2013). Statistical power analysis for the behavioral sciences. Routledge.

[CR20] Dantzer, R., O’Connor, J. C., Freund, G. G., Johnson, R. W., & Kelley, K. W. (2008). From inflammation to sickness and depression: When the immune system subjugates the brain. *Nature Reviews Neuroscience*, *9*(1), 46–56. 10.1038/nrn2297 18073775 10.1038/nrn2297 PMC2919277

[CR21] De Groot, A., Koot, H. M., & Verhulst, F. C. (1994). Cross-cultural generalizability of the Child Behavior Checklist cross-informant syndromes. *Psychological Assessment*, *6*(3), 225–230. 10.1037/1040-3590.6.3.225

[CR22] Dominguez-Bello, M. G., Costello, E. K., Contreras, M., Magris, M., Hidalgo, G., Fierer, N., & Knight, R. (2010). Delivery mode shapes the acquisition and structure of the initial microbiota across multiple body habitats in newborns. *Proceedings of the National Academy of Sciences*, *107*(26), 11971–11975. 10.1073/pnas.1002601107 10.1073/pnas.1002601107 PMC2900693 20566857

[CR23] Drexhage, H. (2023). *Immuno-psychiatrie* (2nd ed.). SWP.

[CR24] Eaton, W. W., Byrne, M., Ewald, H., Mors, O., Chen, C. Y., Agerbo, E., & Mortensen, P. B. (2006). Association of schizophrenia and autoimmune diseases: linkage of Danish national registers. *American Journal of Psychiatry*, *163*(3), 521–528. 10.1176/appi.ajp.163.3.521 16513876 10.1176/appi.ajp.163.3.521

[CR25] García-Marcos, L., Asher, M. I., Pearce, N., Ellwood, E., Bissell, K., Chiang, C. Y., et al. (2022). The burden of asthma, hay fever, and eczema in children in 25 countries: GAN Phase I study. *European Respiratory Journal*, *60*(3), 2102866. 10.1183/13993003.02866-2021 35144987 10.1183/13993003.02866-2021 PMC9474895

[CR26] Hsiao, E. Y. (2013). Immune dysregulation in autism spectrum disorder. *International Reviews Neurobiology*, *113*, 269–302. 10.1016/B978-0-12-418700-9.00009-5 10.1016/B978-0-12-418700-9.00009-5 24290389

[CR27] Hu, C., Nijsten, T., Pasmans, S. G. M. A., De Jongste, J. C., Jansen, P. W., & Duijts, L. (2020). Associations of eczema phenotypes with emotional and behavioural problems from birth until school age: The Generation R Study. *British Journal of Dermatology*, *183*(2), 311–320. 10.1111/bjd.18762 31730242 10.1111/bjd.18705 PMC7496612

[CR28] Infante, M., Slattery, M. J., Klein, M. H., & Essex, M. J. (2007). Association of internalizing disorders and allergies in a child and adolescent psychiatry clinical sample. *Journal of Clinical Psychiatry*, *68*(9), 1419–1425. 10.4088/JCP.v68n0910 17915983 10.4088/jcp.v68n0915

[CR29] Jeppesen, R., & Benros, M. E. (2019). Autoimmune diseases and psychotic disorders. *Frontiers in Psychiatry*, *10*, 131. 10.3389/fpsyt.2019.00131 30949074 10.3389/fpsyt.2019.00131 PMC6435494

[CR30] Khandaker, G. M., Cousins, L., Deakin, J., Lennox, B. R., Yolken, R., & Jones, P. B. (2015). Inflammation and immunity in schizophrenia: implications for pathophysiology and treatment. *Lancet Psychiatry*, *2*(3), 258–270. 10.1016/S2215-0366(14)00122-9 26359903 10.1016/S2215-0366(14)00122-9 PMC4595998

[CR31] ​Klumper, J., Ravelli, A. C. J., Roos, C., Abu-Hanna, A., & Oudijk, M. A. (2024). Trends in preterm birth in the Netherlands in 2011–2019: A population-based study among singletons and multiples. *Acta Obstetricia et Gynecologica Scandinavia*, *103*(3), 449–458. 10.1111/aogs.14684 10.1111/aogs.14684 PMC10867384 37904587

[CR62] Knopik, V. S. (2009). Maternal smoking during pregnancy and child outcomes: Real or spurious effect?. *Developmental Neuropsychology, 34*(1), 1–36. 10.1080/87565640802564366 10.1080/87565640802564366 PMC3581055 19142764

[CR32] Kurth, S., Olini, N., Huber, R., & LeBourgeois, M. (2015). Sleep and early cortical development. *Current Sleep Medicine Reports*, *1*, 64–73. 10.1007/s40675-015-0027-6 26807347 10.1007/s40675-014-0002-8 PMC4721216

[CR33] Lam, M., Chen, C. Y., Li, Z., Martin, A. R., Bryois, J., Ma, X., & Huang, H. (2019). Comparative genetic architectures of schizophrenia in East Asian and European populations. *Nature Genetics*, *51*(12), 1670–1678. 10.1038/s41588-019-0512-x 31740837 10.1038/s41588-019-0512-x PMC6885121

[CR34] Leboyer, M., Oliveira, J., Tamouza, R., & Groc, L. (2016). Is it time for immunopsychiatry in psychotic disorders? *Psychopharmacology (Berl)*, *233*, 1651–1660. 10.1007/s00213-016-4266-1 26988846 10.1007/s00213-016-4266-1

[CR35] Lowe, A. J., Carlin, J. B., Bennett, C. M., et al. (2007). The natural history of allergic disease: An Australian cohort study. *Pediatric Allergy and Immunology*, *18*(8), 751–757.

[CR36] Majde, J. A., & Krueger, J. M. (2005). Links between the innate immune system and sleep. *Journal of Allergy and Clinical Immunology*, *116*(6), 1188–1198. 10.1016/j.jaci.2005.08.005 16337444 10.1016/j.jaci.2005.08.005

[CR38] Marques, A. H., O’Connor, T. G., Roth, C., Susser, E., & Bjørke-Monsen, A. L. (2013). The influence of maternal prenatal and early childhood nutrition and maternal prenatal stress on offspring immune system development and neurodevelopmental disorders. *Frontiers in Neuroscience*, *7*, 120. 23914151 10.3389/fnins.2013.00120 PMC3728489

[CR37] Mayer, E. A., Tillisch, K., & Gupta, A. (2015). Gut/brain axis and the microbiota. *The Journal of Clinical Investigation*, *125*(3), 926–938. 10.1172/JCI76304 25689247 10.1172/JCI76304 PMC4362231

[CR39] Miravitlles, M., Andreu, I., Romero, Y., Sitjar, S., Altés, A., & Anton, E. (2012). Difficulties in differential diagnosis of COPD and asthma in primary care. *British Journal of General Practice*, *62*(595), e68–e75. 10.3399/bjgp12X625073 10.3399/bjgp12X625111 PMC3268496 22520766

[CR40] Nanda, M. K., LeMasters, G. K., Levin, L., Rothenberg, M. E., Assa’ad, A. H., Newman, N., & Ryan, P. H. (2016). Allergic diseases and internalizing behaviors in early childhood. *Pediatrics*, *137*(1), e20152016. 10.1542/peds.2015-2016 10.1542/peds.2015-1922 PMC4702018 26715608

[CR41] Nielsen, P. R., Benros, M. E., & Dalsgaard, S. (2017). Associations between autoimmune diseases and attention-deficit/hyperactivity disorder: a nationwide study. *Journal of the American Academy of Child and Adolescent Psychiatry*, *56*(3), 234–240. 10.1016/j.jaac.2016.12.010 28219489 10.1016/j.jaac.2016.12.010

[CR42] Ohuma, E. O., Moller, A. B., Bradley, E., Chakwera, S., Hussain-Alkhateeb, L., Lewin, A., & Moran, A. C. (2023). National, regional, and global estimates of preterm birth in 2020, with trends from 2010: A systematic analysis. *Lancet*, *402*(10409), 1261–1271. 10.1016/S0140-6736(23)01548-4 37805217 10.1016/S0140-6736(23)00878-4

[CR43] Pariante, C. M. (2017). Why are depressed patients inflamed? A reflection on 20 years of research on depression, glucocorticoid resistance and inflammation. *European Neuropsychopharmacology*, *27*(6), 554–559. 10.1016/j.euroneuro.2017.04.001 28479211 10.1016/j.euroneuro.2017.04.001

[CR44] Patel, S., Cooper, M. N., Jones, H., Whitehouse, A. J. O., Dale, R. C., & Guastella, A. J. (2021). Maternal immune-related conditions during pregnancy may be a risk factor for neuropsychiatric problems in offspring throughout childhood and adolescence. *Psychological Medicine*, *51*(16), 2904–2914. 10.1017/S0033291720001580 32476637 10.1017/S0033291720001580

[CR46] Pieters, S., Van Der Vorst, H., Burk, W. J., Wiers, R. W., & Engels, R. C. (2010). Puberty-dependent sleep regulation and alcohol use in early adolescents. *Alcoholism: Clinical and Experimental Research*, *34*(9), 1512–1518. 20569245 10.1111/j.1530-0277.2010.01235.x

[CR45] Pieters, S., Burk, W. J., Van der Vorst, H., Dahl, R. E., Wiers, R. W., & Engels, R. C. (2015). Prospective relationships between sleep problems and substance use, internalizing and externalizing problems. *Journal of Youth and Adolescence*, *44*, 379–388. 10.1007/s10964-014-0212-z 25385390 10.1007/s10964-014-0213-9

[CR47] Sandall, J., Tribe, R. M., Avery, L., Mola, G., Visser, G. H., Homer, C. S. E., & Gibbons, D. (2018). Short-term and long-term effects of caesarean section on the health of women and children. *The Lancet*, *392*(10155), 1349–1357. 10.1016/S0140-6736(18)31930-5 10.1016/S0140-6736(18)31930-5 30322585

[CR49] Sevelsted, A., Stokholm, J., & Bisgaard, H. (2016). Risk of asthma from cesarean delivery depends on membrane rupture. *Journal of Pediatrics*, *171*, 38–42e4. 10.1016/j.jpeds.2015.12.066 26825289 10.1016/j.jpeds.2015.12.066

[CR48] Stokholm, J., Blaser, M. J., Thorsen, J., Rasmussen, M. A., Waage, J., Vinding, R. K., & Bisgaard, H. (2018). Maturation of the gut microbiome and risk of asthma in childhood. *Nature Communications*, *9*(1), 141. 10.1038/s41467-017-02431-4 29321519 10.1038/s41467-017-02573-2 PMC5762761

[CR50] Suh, D. I., Chang, H. Y., Lee, E., Yang, S. I., & Hong, S. J. (2017). Prenatal maternal distress and allergic diseases in offspring: Review of evidence and possible pathways. *Allergy Asthma & Immunology Research*, *9*(3), 200–211. 10.4168/aair.2017.9.3.200 10.4168/aair.2017.9.3.200 PMC5352571 28293926

[CR51] Thamm, R., Poethko-Müller, C., Hüther, A., & Thamm, M. (2018). Allergic diseases in children and adolescents in Germany. Results of the cross-sectional KiGGS Wave 2 study and trends. *Journal of Health Monitoring*, *3*(3), 3–16. 10.17886/RKI-GBE-2018-080 35586803 10.17886/RKI-GBE-2018-082 PMC8848849

[CR52] Van der Mheen, M., Zijlmans, J., van der Doelen, D. M., Klip, H., van der Lans, R. M., Ruisch, I. H., & Polderman, T. J. C. (2024). Patterns of Mental Disorders in a Large Child Psychiatric Sample (N = 65,363): A DREAMS Study. *JAACAP Open*. 10.1016/j.jaacop.2024.06.007 41367955 10.1016/j.jaacop.2024.06.007 PMC12684454

[CR53] Van Gent, T., Heijnen, C. J., & Treffers, P. D. (1997). Autism and the immune system. *Journal of Child Psychology and Psychiatry*, *38*(3), 337–349. 10.1111/j.1469-7610.1997.tb01518.x 9232480 10.1111/j.1469-7610.1997.tb01518.x

[CR54] Van Tetering, E. M. A., Mies, G. W., Klip, H., Pillen, S., Muskens, J. B., Polderman, T. J. C., Van der Mheen, M., Staal, W. G., & Pieters, S. (2024). The relationship between sleep difficulties and externalizing and internalizing problems in children and adolescents with mental illness. *Journal of Sleep Research*, *e14398*. 10.1111/jsr.14398 10.1111/jsr.14398 PMC12069754 39533513

[CR55] Verkleij, M., van de Griendt, E. J., Kaptein, A. A., van Essen-Zandvliet, L., Duiverman, E., & Geenen, R. (2011). Behavioral problems in children and adolescents with difficult-to-treat asthma. *Journal of Asthma*, *48*(1), 18–24. 10.3109/02770903.2010.536981 10.3109/02770903.2010.528497 21043987

[CR56] Von Mutius, E. (2009). The rising trends in asthma and allergic disease. *PLoS Medicine*, *6*(6), e1000141. 9988447 10.1046/j.1365-2222.1998.028s5045.x

[CR57] Walker, R. W., Clemente, J. C., Peter, I., & Loos, R. J. (2017). The prenatal gut microbiome: are we colonized with bacteria in utero? *Pediatric Obesity*, *12*, 3–17. 10.1111/ijpo.12217 28447406 10.1111/ijpo.12217 PMC5583026

[CR58] Willemsen, G., Van Beijsterveldt, T. C., Van Baal, C. G., Postma, D., & Boomsma, D. I. (2008). Heritability of self-reported asthma and allergy: a study in adult Dutch twins, siblings and parents. *Twin Research and Human Genetics*, *11*(2), 132–142. 10.1375/twin.11.2.132 18361713 10.1375/twin.11.2.132

[CR59] World Health Organization (WHO) (2021). *Caesarean section rates continue to rise, amid growing inequalities in access*. WHO. Retrieved from: https://www.who.int/news/item/16-06-2021-caesarean-section-rates-continue-to-rise-amid-growing-inequalities-in-access

[CR60] Zablotsky, B., Black, L. I., & Akinbami, L. J. (2023). Diagnosed allergic conditions in children aged 0–17 years: United States, 2021. *Nchs Data Brief*, (459), 1–8. 10.15620/cdc:123250 36700870

