# Supplementary material for: Perinatal Risk Factors, Allergic Conditions and Psychosocial Problems in Children and Adolescents with Mental Health Problems
Source: Res Child Adolesc Psychopathol. 2026 May 26;54(3):71. doi: 10.1007/s10802-026-01465-8 (PMC13212645; doi:10.1007/s10802-026-01465-8)
Supplement: Supplementary file 1 — Supplementary Material 1 [file 10802_2026_1465_MOESM1_ESM.docx]

**Supplementary Table 1**

*Cumulative Effects on Internalizing Problems*

|  | *B* | *SE* | *p* |
| --- | --- | --- | --- |
| Age | .98 | .08 | < .001 |
| Sex | 5.00 | .51 | < .001 |
| Allergic conditions | 2.18 | .81 | .007 |
| Perinatal risk factors | .10 | .55 | .851 |
| Allergic conditions (squared) | .01 | .17 | .957 |
| Perinatal risk factors (squared) | .17 | .21 | .422 |
| Interaction allergic conditions x perinatal risk factors | -.13 | .26 | .611 |

**Supplementary Table 2**

*Specific Effects on Internalizing Problems*

|  | *B* | *SE* | *p* |
| --- | --- | --- | --- |
| Age | 1.00 | .08 | < .001 |
| Sex | 4.94 | .51 | < .001 |
| Asthma | 1.36 | 1.25 | .279 |
| Hay Fever | 1.13 | .80 | .160 |
| Eczema | 3.17 | .94 | < .001 |
| Food allergy | 3.67 | 1.22 | .003 |
| Preterm birth | -.20 | .90 | .826 |
| Low birth weight | .32 | .92 | .730 |
| C-section | 1.04 | .66 | .117 |
| Maternal prenatal illness or infections | 3.38 | 1.39 | .015 |

**Supplementary Table 3**

*Cumulative Effects on Externalizing Problems*

|  | *B* | *SE* | *p* |
| --- | --- | --- | --- |
| Age | -.29 | .08 | < .001 |
| Sex | -2.33 | .51 | < .001 |
| Allergic conditions | -.67 | .81 | .411 |
| Perinatal risk factors | .11 | .56 | .842 |
| Allergic conditions (squared) | .31 | .17 | .073 |
| Perinatal risk factors (squared) | -.01 | .21 | .976 |
| Interaction allergic conditions x perinatal risk factors | .22 | .26 | .396 |

**Supplementary Table 4**

*Specific Effects on Externalizing Problems*

|  | *B* | *SE* | *p* |
| --- | --- | --- | --- |
| Age | -.28 | .08 | < .001 |
| Sex | -2.35 | .51 | < .001 |
| Asthma | -1.38 | 1.26 | .272 |
| Hay Fever | .78 | .80 | .331 |
| Eczema | 1.80 | .95 | .058 |
| Food allergy | -.15 | 1.23 | .903 |
| Preterm birth | -.19 | .91 | .838 |
| Low birth weight | -.55 | .93 | .555 |
| C-section | .57 | .66 | .388 |
| Maternal prenatal illness or infections | 3.41 | 1.39 | .015 |
